# Detection of Pathogenic *Leptospira* in Captive Chelonians (*Kinosternon scorpioides*—Linnaeus, 1766) in the Brazilian Amazon

**DOI:** 10.3390/ani14091334

**Published:** 2024-04-29

**Authors:** Rafael Souza Freitas, Katarine de Souza Rocha, Louysse Helene Monteiro, Thais Fernandes Alexandre, Thamillys Rayssa Marques Monteiro, Betsy Emely Tavares Honorio, Mayra Coelho Gripp, Claudio Douglas de Oliveira Guimarães, Maria das Dores Correia Palha, Thamirys de Souza Gonçalves, Alessandra Scofield, Carla Cristina Guimarães de Moraes

**Affiliations:** 1 Laboratory of Zoonoses and Public Health (LZSP), Graduate Program in Animal Health in the Amazon, Institute of Veterinary Medicine, Federal University of Pará (UFPA), Castanhal 68743-970, PA, Brazil; katarinemv@gmail.com (K.d.S.R.); helenelouysse@gmail.com (L.H.M.); thais.andes19@gmail.com (T.F.A.); thamillysmonteiro@gmail.com (T.R.M.M.); betsyemely@gmail.com (B.E.T.H.); 2 Laboratory of Zoonoses and Public Health (LZSP), Institute of Veterinary Medicine, Federal University of Pará (UFPA), Castanhal 68743-970, PA, Brazil; grippmayra@gmail.com; 3 Socio-Environmental and Water Resources Institute, Federal Rural University of the Amazon, Belém 66077-830, PA, Brazil; cdoguimaraes@gmail.com (C.D.d.O.G.); faunaufra@gmail.com (M.d.D.C.P.); 4 Laboratory of Animal Health, Graduate Program in Animal Health in the Amazon, Federal University of Pará (UFPA), Castanhal 68743-970, PA, Brazil; thamirysgoncalves33@gmail.com (T.d.S.G.); ascofield@ufpa.br (A.S.); ccmoraes@ufpa.br (C.C.G.d.M.)

**Keywords:** environment, leptospirosis, one health, reptiles, reservoirs

## Abstract

**Simple Summary:**

The expansion of human activities into natural areas increases contact between humans, domestic animals, and wildlife, which can facilitate the circulation of infectious agents between these species, leading to the emergence of zoonoses. Several studies have investigated the role of animals, including reptiles, as possible carriers of the *Leptospira* bacterium. This study aimed to detect the DNA of the bacterium in *Kinosternon scorpioides* turtles kept in captivity in a region of the Brazilian Amazon. Blood, cloacal fluid, cloacal lavage, and stomach lavage samples were collected from 40 turtles. Of these, 40% of the animals tested positive for *Leptospira*. Genetic analysis confirmed the identification of the bacteria, which could pose a risk to public health. Handling infected animals could increase the risk of transmitting the disease, especially considering that turtle meat is consumed in the region. This study is the first to detect *Leptospira* in the blood of chelonians, confirming exposure to the pathogen. Although the turtles showed no abnormal clinical signs, it is possible that the clinical signs are unknown in reptiles. In conclusion, captive *K. scorpioides* turtles have been exposed to *Leptospira*.

**Abstract:**

Leptospirosis is a zoonosis of great importance for One Health. In this context, the Amazonian biome may harbor numerous hosts for *Leptospira* spp. that contribute to the maintenance of the pathogen in the environment. Some reptiles, such as chelonians, have been little studied in terms of their involvement with leptospires. The objective of this study was to detect *Leptospira* spp. DNA in *Kinosternon scorpioides* turtles kept in captivity in a region of the Brazilian Amazon. A total of 147 samples of blood (*n* = 40), cloacal fluid (*n* = 27), cloacal lavage (*n* = 40), and stomach (*n* = 40) were collected from 40 chelonians. After DNA extraction, the samples were subjected to amplification of a 331 base pair product of the 16S rRNA gene using the Lep1 and Lep2 primers. PCR products were Sanger sequenced, assembled, and subjected to online blast search and phylogenetic analysis. Of the animals tested, 40% (16/40, 95% confidence interval [CI]: 25–55) had at least one or two samples positive for *Leptospira* spp. Considering the total number of samples collected, 12.93% (19/147) were positive, being blood clots (27.5%; 11/40), followed by cloacal washings (10%; 4/40), cloacal fluid (11.11%; 3/27) and gastric washings (2.5%; 1/40). Of these, 11 samples were sequenced and showed 99% to 100% identity with *Leptospira interrogans* sequences, which was confirmed by phylogenetic analysis. This is the first study to detect pathogenic *Leptospira* DNA in chelonians in a region of the Brazilian Amazon. It has been concluded that *K. scorpioides* turtles in captivity have been exposed to pathogenic *Leptospira*.

## 1. Introduction

Leptospirosis is an important anthropozoonosis for public health because its epidemiological chain involves bacteria, hosts, and the environment [1,2,3]. Despite being a zoonosis with a global impact, its severity is underestimated in Brazil due to the incorrect completion of disease reporting forms, underreporting, misdiagnosis, and stigmatization of the disease associated with poverty and lack of basic sanitation [4,5].

In humans, it’s a systemic disease that can occur acutely and is characterized by an icterohemorrhagic syndrome. However, it presents variable clinical signs such as fever, vomiting, and diarrhea, affecting organs such as the lungs, intestines, liver, and kidneys [6]. In production animals, the infection causes reproductive problems, resulting in significant animal and economic losses [7]. Furthermore, in hosts such as dogs, the same pathogenic strain can exhibit varying degrees of severity, ranging from asymptomatic infections to fatal cases [8].

This zoonosis is caused by pathogenic species of *Leptospira* spp. of the order *Spirochaetales*, family *Leptospiraceae*. The genus consists of Gram-negative bacteria with a spiral or helical morphology and periplasmic flagella that provide them with great mobility. These spirochetes are 0.1 to 0.2 µm in diameter and 6 to 12 µm long, and their membrane is full of lipopolysaccharides (LPS) that are unique to each serovar [6].

Previously, only *Leptospira interrogans* and *Leptospira biflexa* represented these bacteria, which were classified as both pathogenic and saprophytic. However, recent phylogenetic analyses of their 16S and 23S rRNA genes resulted in their reclassification into pathogenic, intermediate, and saprophytic species [9]. In 2019, another classification was suggested, dividing the genus into pathogenic (P1 and P2) and saprophytic (S1 and S2) species [10].

The pathogenic species exhibit distinct characteristics. For instance, in vitro experiments demonstrated that these leptospires could maintain their virulence and survive even after being stored for 20 months in cold, acidic, and nutrient-poor water [11]. Furthermore, these bacteria are autotrophic in B12 (cyanocobalamin), possess proteins capable of modifying their virulence, and express specific genes, such as LipL32 and LigB [10,12,13].

In mammals, infection occurs through direct horizontal transmission via contact with the urine, blood, or tissues of infected animals, or indirectly through contaminated materials, food, or the environment with pathogenic species [14,15]. A clinical picture results from two phases: infection/multiplication (blood circulation) and dissemination in the urine [3]. These phases can significantly impact the diagnosis.

Molecular identification can improve the speed and sensitivity of both phases of infection. In contrast, dark-field microscopy requires a minimum of 10 spirochetes/mL in the urine for adequate visualization. However, the most reliable method is the microscopic agglutination test (MAT). This test relies on the production of antibodies by the host, the specificity of the circulating serovars, and the collection of antigens used to perform the test [16,17].

These methods are widely used in human and companion animal clinical practice. They are also being used to identify other *Leptospira* reservoirs around the world. These discoveries have been made mainly in South America due to its high biodiversity, but also in the USA, Italy, the Netherlands, Japan, and Madagascar. The newly identified hosts are distributed across Carnivora, Didelphimorphia, Rodentia, Cetacea, Cingulata, Afrosoricida, Chiroptera, Primates, Reptilia, and Amphibia [18].

The expansion of human activities into natural environments can result in increased contact between humans, domestic animals, and wildlife, potentially leading to the transmission of zoonotic agents [5,19]. Therefore, researchers are investigating the role of unknown hosts, including chelonians [20,21,22,23,24,25,26,27,28,29,30], as potential *Leptospira* reservoirs.

Reptiles, including chelonians, can host various etiological agents, increasing the maintenance and spread of zoonotic agents such as *Leptospira* [22]. Furthermore, breeding wild animals as pets, cultural practices, such as the consumption of bushmeat obtained through hunting, illegal breeding for trade, and, primarily, a lack of management knowledge are risk factors associated with infection [31].

Serological investigations have been conducted on chelonians in the United States and Italy, revealing varying seroprevalences of anti-*Leptospira* antibodies [22,23,24,25]. Similarly, antibodies were detected in the Brazilian Amazon turtles of the *Podocnemis unifilis*, *Podocnemis expansa*, and *Rhinoclemmys punctularia* species [26,27,28].

Direct diagnostic methods such as culture, dark field microscopy, and PCR are crucial for confirming the epidemiological significance of chelonians in maintaining leptospires in the environment [32]. A single published study conducted in Brazil reported the amplification of leptospiral DNA extracted from chelonian samples [29]. Globally, investigations of *Leptospira* in these animals at the molecular level [20,21,22,29,30], as well as the genetic sequencing of positive samples, have been insufficient; as an example, only two studies confirmed the results through sequencing [21,30].

In addition, there is a paucity of research on the identification of *Leptospira* spp. in chelonians. Even in those studies where anti-*Leptospira* antibodies were detected along with leptospiral DNA [21,22,29], the animals did not show clinical signs of leptospirosis or abnormal clinical signs. The available evidence does not document how the bacterium acts in the organisms of these animals [32].

The Amazonian biome contains a variety of animals that have not been tested for the presence of *Leptospira* spp. One of these animals is the scorpion mud turtle (*Kinosternon scorpioides*), also known as Muçuã or Jurará, a small semiaquatic freshwater turtle found from Costa Rica to northern Argentina and Brazil. Due to their semiaquatic behavior, these animals may play a critical role in the epidemiology of the pathogen [33,34,35].

Human intervention in the Brazilian Amazon underscores the necessity of identifying poorly researched *Leptospira* hosts and analyzing their effects on the environment. Therefore, the aim of this research was to detect *Leptospira* spp. DNA in *K. scorpioides* samples at an Amazon biome conservation breeding site. The hypotheses that were tested were as follows: (1) chelonians in captivity may be exposed to the bacterium *Leptospira* spp.; and (2) in addition to harboring leptospires in fluids such as cloacal lavage, cloacal fluid, and gastric lavage, chelonians would also harbor the bacterium in their blood.

## 2. Materials and Methods

### 2.1. Study Area and Animals

**Figure 1:**
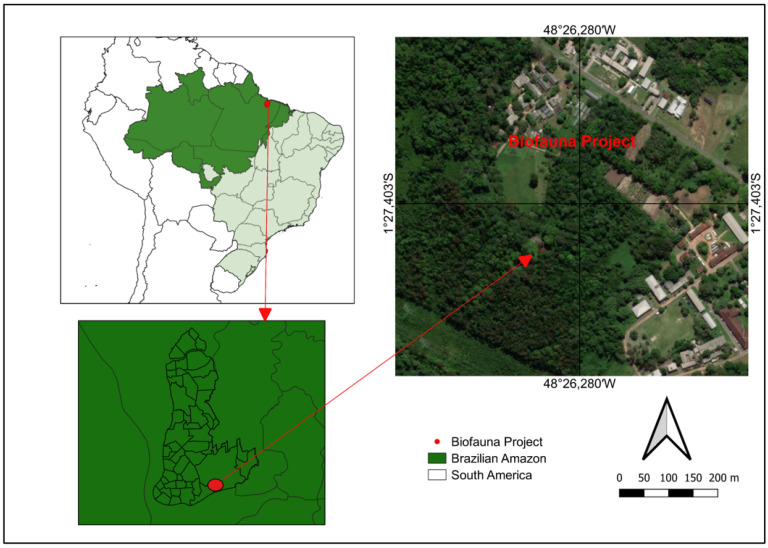
Map (QGIS 3.22.3) showing the location of the Brazilian Amazon biome in South America, the study area within the Amazon biome, and the location of the chelonian captivity. The satellite image shows the forest fragment within the Federal Rural University of Amazonia and the location of the captive breeding facility surrounded by vegetation. The arrow indicates the location where the animal samples were taken, and where the biofauna project is being carried out.

This study was carried out with *K. scorpioides* chelonians kept in captivity in a conservation breeding facility at the main campus of the Federal Rural University of Amazonia (UFRA) in Belém, State of Pará (1°27′26.7″ S 48°26′19.7″ W), in the area assigned to the Bio-Fauna Project of the Socioenvironmental and Water Resources Institute (ISARH)/UFRA, Belém, PA, Brazil, during four expeditions conducted in 2019. The animals are part of the stock of the Bio-Fauna Project (Figure 1) and were captured by the Brazilian Institute of Environment and Renewable Natural Resources (IBAMA), PA, Brazil.

Because they were captured, there is no information on the origin or age of the chelonians used in this study. On the other hand, the duration of captivity of each specimen was not considered, as the specimens lived together for at least one year. Animals were handled daily for feeding, water changes, monitoring, and data collection. In addition, the animals lived in an outdoor water-storage-tank-type enclosure with environmental enrichment to mimic the natural environment.

### 2.2. Sample Collection and Processing

All the chelonians present in the enclosures were sampled, and a total of 40 animals and 147 biological samples were obtained (whole blood, *n* = 40; gastric lavage, *n* = 40; cloacal lavage, *n* = 40; and cloacal fluid, *n* = 27). All the samples collected were stored in isothermal boxes and transported to the Laboratory of Zoonoses and Public Health of the Federal University of Pará (UFPA), Castanhal Campus, for processing.

**Figure 2:**
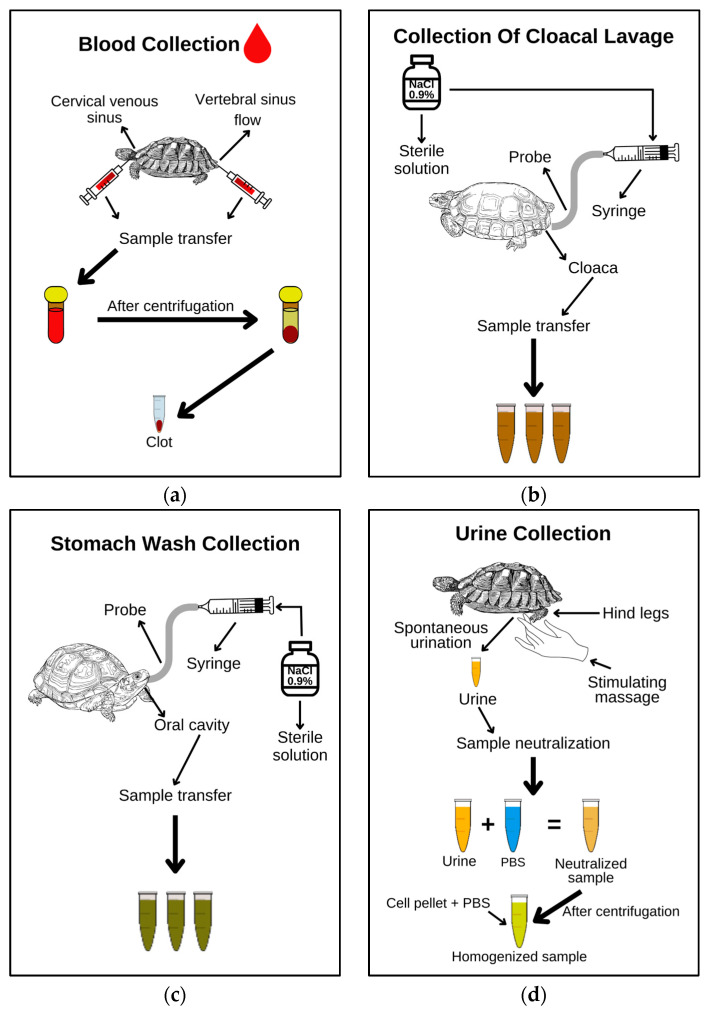
Flowchart showing collection and processing of blood clots (**a**), cloacal lavages (**b**), stomach lavages (**c**), and urine (**d**) samples from *K. scorpioides* in the Biofauna Project, municipality of Belém, Pará State, Brazil.

Whole-blood samples were collected aseptically by puncture of the coccygeal vein and cervical sinus using needles (25 × 0.7 mm) and 3 mL disposable syringes into amber microtubes containing a separation gel (BD Microtainer^®^, Franklin Lakes, NJ, USA). After centrifugation, the serum was removed, and the clots were used for DNA sequencing (Figure 2a).

Cloacal contents were obtained by washing according to the methods of another study conducted in Brazil [29], with modifications. Disposable 20 mL syringes, urethral probe no. 6, sterile 0.9% sodium chloride (NaCl) solution, and 15 mL polypropylene conical bottom tubes were used (Figure 2b).

The stomach contents were also determined by washing with NaCl (Figure 2c) [29]. However, the distance between the oral cavity and the gastric opening was measured by the external part of the plastron, which marked the probe for each animal. This procedure was designed to avoid injury during harvesting, both because the diameter of the probe could damage the animal’s esophagus and because its size could damage the gastric mucosa. Hemostatic forceps were used to keep the oral cavity open. A premarked probe of the appropriate size was then inserted into each animal, a syringe was attached to the other end of the probe, and 20 mL of sterile 0.9% sodium chloride (NaCl) solution was administered.

Chemical restraint was not necessary for this stomach washing procedure because it is not considered invasive [36]; the animals tend to be cooperative due to their anatomy and the whole procedure was carried out quickly considering the animals’ physical well-being, with the procedure lasting a maximum of 3 min for each animal.

Cloacal fluid was collected by massaging the hind legs near the right and left femoral shields of the plastron to induce urination directly into sterile 15 mL conical bottom tubes (Figure 2d). These samples were immediately neutralized with phosphate-buffered saline (PBS) (pH 7.2) at a ratio of 2.5:1 (cloacal fluid–PBS) [37]. After centrifugation at 5200 rpm for 15 min, the supernatant was discarded, and the cell pellet was transferred to a 1.5 mL microtube containing 1 mL of PBS (pH 7.2). A second centrifugation was performed at 10,000 rpm for 2 min, the supernatant was discarded, and the cell pellet was resuspended in 200 μL of PBS (pH 7.2).

All the samples collected were stored at −20 °C until DNA was extracted for subsequent molecular analysis.

### 2.3. DNA Extraction

For DNA extraction, cloacal fluid, gastric wash, cloacal wash, and blood clot samples were extracted using the IllustraTM Tissue and Cell Prep Mini Spin Kit (GE Healthcare, Buckinghamshire, UK) according to the manufacturer’s instructions. DNA was extracted in the same year of collection and the integrity of the extraction was assessed by agarose gel electrophoresis.

### 2.4. Molecular Analysis

To detect *Leptospira* spp. DNA, we performed the polymerase chain reaction (PCR) using the primers Lep1 (5′GGCGGCGCGTCTTAAACATG3′) and Lep2 (5′TTCCCCCCATTGAGCAAGATT3′), which amplify a 331 base pair (bp) fragment of the 16S rRNA gene [38] The PCR mix and thermal cycling steps were carried out according to a protocol previously described [37].

All reaction mixtures included two positive controls and one contamination control. The positive controls were DNA extracted from isolates of *Leptospira* serovar Icterohaemorrhagiae and Patoc obtained from an EMJH (Ellinghausen, McCullough, Johnson, and Harris) culture medium, while the contamination control was ultrapure water with no DNA added to the amplification solution.

All the reactions were run on a gradient thermal cycler (Veriti 96 Well Thermal Cycler, Applied Biosystems^®^, Foster City, CA, USA). The PCR products were subjected to horizontal electrophoresis on a 1.5% agarose gel and stained with Gel Red^®^ (Biotium™, Fremont, CA, USA). Bands of the expected size were visualized under ultraviolet light in a transilluminator (Gel DOCTM XR+ Imaging System Bio-Rad, Hercules, CA, USA) with a photo documentation system (Image Lab™ V. 5.2-Bio-Rad).

Amplification products with the best electrophoresis bands were selected to be sequenced in commercial facilities, totaling 11 samples (ATCGene Análises Molecularis Ltd.a., Alvorada, RS, Brazil). for sequencing using a commercial kit (ExoSAP-IT™ PCR Product Cleanup Reagent, Applied Biosystems^®^) and an automated sequencer (ABI Prism 3500 Genetic Analyzer, Applied Biosystems^®^).

The genetic sequences obtained from sequencing were processed using BioEdit Sequence Alignment Editor software (Version 7.7). Consensus sequences were generated for each sample amplified by the forward and reverse primers, with total lengths ranging from 265 bp to 338 bp. The consensus sequences were compared with sequences from BLAST (Basic Local Alignment Search Tool—www.blast.ncbi.nlm.nih.gov (accessed on 12 February 2024), USA) to determine the percentage identity of the nucleotides.

The consensus sequences were aligned using the AliView v.1.18 program [39] and the MAFFT alignment algorithm [40]. The *Leptospira* sequences from the P1, P2, and S1 groups were added from the Genbank database, selecting those with the closest identities to the consensus sequence. The resulting sequences were submitted to the GenBank database.

For phylogenetic analysis, 26 reference sequences from different *Leptospira* species with the closest identity to the consensus sequences were selected (*n* = 11), and one sequence from *Leptonema illini* was chosen as the outgroup. The sequences ranged in size from 674 bp to 1516 bp but were trimmed to a similar size (338 bp) before analysis.

The Jmodeltest v.2.0 algorithm [41,42] was used to calculate the evolutionary models best suited to the data. The phylogenetic relationships were built using the Neighbor-Joining (NJ) method with 1000 bootstrap replicates, and support statistic values below 70% were disregarded. The MEGA v.6.0 program [43] was employed for this purpose.

## 3. Results

Considering the number of animals analyzed, 40% (16/40, 95% confidence interval [CI]: 25–55) of the chelonians had at least one or two positive samples for *Leptospira* spp. (Table 1).

Among the samples collected, 12.93% (19/147) were positive (Table 2). A greater frequency of *Leptospira* spp. DNA was detected in blood clots (27.5%; 11/40), followed by cloacal washings (10%; 4/40), cloacal fluid (11.11%; 3/27), and gastric washings (2.5%; 1/40).

In the BLASTn analysis, the sequenced samples (*n* = 11) showed 99–100% maximum identity with *Leptospira interrogans* sequences from isolates in different countries. The assembled nucleotides have been deposited in GenBank (http://www.ncbi.nlm.nih.gov (accessed on 12 February 2024), USA) under access numbers OP312971 to OP312981.

**Figure 3:**
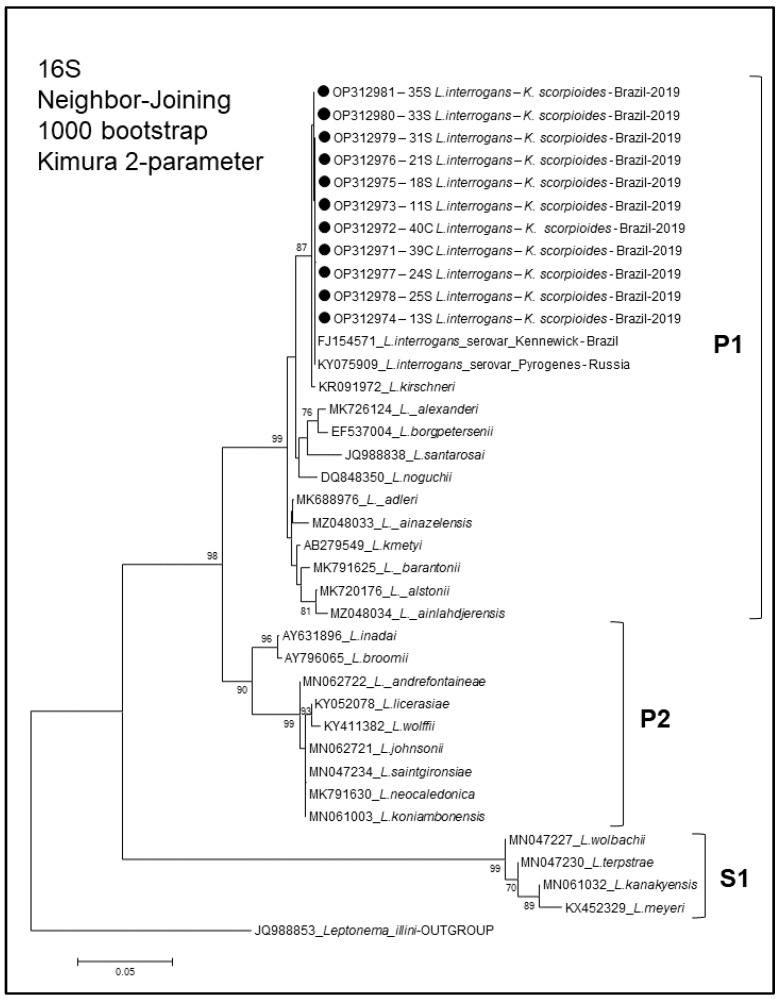
Phylogenetic tree constructed using the neighbor-joining (K2P) method with paired 16S rRNA gene sequences of *Leptospira* spp. isolates, showing only bootstrap values > 70%. The sequences obtained from eleven samples of *Kinosternon scorpioides* are marked by a black period. The sequence of one isolate from *Leptonema illini* (JQ988853) was used as an outgroup.

Phylogenetic analysis confirmed the identity of the partial sequences of the 16S rRNA gene of *Leptospira* spp. The sequences obtained from the *K. scorpioides* samples were grouped in the same clade as the sequences of the *L. interrogans* serovar Kennewick from Brazil (FJ154571) and *L. interrogans* serovar Pyrogenes from Russia (KY075909) within the subclade of *Leptospira*, which form the P1 group of pathogenic species (Figure 3).

## 4. Discussion

This is the first report of natural exposure and detection of *Leptospira interrogans* DNA in chelonians kept in captivity in a region of the Amazon biome and in the species *K. scorpioides*.

This study is the first of its kind to detect *Leptospira* DNA in the blood of chelonians. Blood collection from chelonians can be challenging due to the rapid coagulation of blood, non-visible vessels, and the impossibility of using a tourniquet. The jugular vein is the preferred route for collection, but due to the strong retraction muscles of the chelonian head, it can be difficult to keep the neck taut without sedation. In this study, the coccygeal vein was preferred for blood collection since the animals were not sedated [44].

The mechanism of adaptation of these bacteria in ectothermic animals, where body temperature is influenced by environmental factors [45], requires further study since leptospires multiply satisfactorily in hosts with stable temperatures, such as mammals.

Previous studies on chelonians have only detected leptospiral DNA in samples of stomach wash, cloacal wash, cloacal swab, cloacal fluid, and kidney [21,22,29,30]. While the use of whole blood from these animals to search for leptospires is not a new practice [20], this study demonstrates that blood clots are also effective in detecting *Leptospira* in chelonians.

The cloaca of these animals consists of three cavities: the urodeum, the coprodeum, and the proctodeum [40]. However, this internal division is not very distinct in these animals; the urine is deposited in the urodeum, but when an animal needs to hydrate, it can return to the bladder or go directly to the proctodeum, where it contacts the animal’s feces and other urogenital fluids [46].

Previous studies have also suggested that chelonians can serve as natural hosts for these bacteria and excrete them for extended periods [29,47]. The absence of leptospires in some samples may be due to the purity of the collected content, as fecal matter and urinary acidity can chelate leptospiral DNA [48]. It is also possible that the bacteria also transiently shed [49].

Although pathogenic *Leptospira* DNA was found in *K. scorpioides* samples, it is only possible to confirm the viability of leptospires using specific culture media such as Fletcher and EMJH (Ellinghausen–McCullough–Johnson–Harris) [50]. Therefore, it cannot be concluded that these animals are spreading these bacteria.

The study utilized conventional PCR, which yielded favorable results compared to other studies conducted in Brazil using the same technique [20,29]. In Italy, nested PCR was used on cloacal swabs, which is considered more sensitive, and *Leptospira* DNA was detected in 20% (10/50) of the samples [21]. Real-time PCR analysis of cloacal swab samples in the U.S. revealed a high prevalence of animals with *Leptospira* DNA (73.5%–25/34) [22].

Our research identified *L. interrogans* as the species belonging to the P1 subgroup based on phylogenetic analysis. Generally, these species have longer genomes and a greater diversity of genes encoding virulence factors, potentially making the P1 subgroup more pathogenic than the P2 subgroup [10].

In this study, none of the chelonians that tested positive by molecular detection showed abnormal clinical signs, which supports the findings of other studies [21,22,29,30] that have investigated *Leptospira* spp. in chelonians. However, it should be noted that these clinical signs may be present in these animals but are currently unknown.

*Leptospira* spp. DNA was detected in the clot and cloacal lavage fluid of animal 11 and in the clot and cloacal fluid of animal 39. This suggests a potential difference in spirochete behavior in chelonians, as the bacteria were found in both the bloodstream and cloacal fluid simultaneously. In contrast, urinary dissemination in mammals occurs when leptospires are lodged in renal tubules [3,6].

The identification of leptospires in aquatic and semiaquatic animals can aid in the recognition of hosts with distinct profiles from those already known. Semiaquatic animals seem to be more susceptible to exposure and dissemination as they traverse various environments, including soil and water [50,51]. Another study supported the hypothesis that leptospires originate in the soil and are transported to bodies of water during heavy rains, despite their common association with aquatic environments [52].

The chelonians studied in this research were kept in open water enclosures, with access to the ground, which indicates that the animals could have been exposed by contact with the soil. It is also possible that the water was contaminated by opportunistic hosts, such as rodents, as the environment surrounding the enclosure could provide access to water, food, and shelter.

The animals are kept in captivity at the Federal Rural University of Amazonia, which is situated near large forest fragments, such as the Camillo Vianna Utinga State Park. These findings suggest that professionals who work with captive reptiles are at risk of contracting infectious agents. Additionally, the presence of free-living animals around these facilities can contribute to the spread of pathogens in the environment [53].

Since they were captured, there is no information on the origin of the chelonians used in this study. On the other hand, the duration of captivity of each specimen was not considered, as the specimens lived together for at least one year. The discovery of *Leptospira* DNA in the studied samples shows that there is a need to study infectious agents, especially those of a zoonotic nature, that may affect captive animals. In addition, the risk factors associated with exposure to these animals and the people who handle them should be investigated.

In the northern region of Brazil, in addition to the factors directly linked to the One Health triad, it is important to consider sociocultural factors in the transmission of pathogens. In the Amazon biome, chelonian meat is consumed for subsistence purposes [33]. The most consumed species are the Amazonian turtle, Arrau River Turtle, (*Podocnemis expansa*), the Tracajá, Yellow-spotted Amazon River Turtle, (*Podocnemis unifilis*), and the Muçuã, Scorpion Mud Turtle, (*Kinosternon scorpioides*) [34]. Therefore, handling the carcasses of animals with *Leptospira* can pose a zoonotic threat.

Environmental degradation also contributes to the circulation of zoonotic agents. The Amazon biome has edaphoclimatic conditions and diverse fauna that can influence the maintenance of pathogens in the environment. Additionally, forest fragmentation and urban encroachment bring humans and wildlife into closer contact. However, the risk of zoonotic spillover in the Amazon region is often underestimated due to a lack of investment in research and underreporting, likely due to the vast size of the biome [54].

Future studies should aim to identify chelonians exposed to *Leptospira* spp. by culturing samples from the stomach lavage, cloacal lavage, cloacal fluid, and blood to determine the viability of the detected agent. This approach may lead to the identification of a serovar that has not yet been described in the Brazilian Amazon. In addition, this work expands the list of reptile hosts of pathogenic *Leptospira*, and the prevalence of positive animals in this study may represent a high risk for their handlers.

## 5. Conclusions

Leptospiral DNA could be detected in cloacal lavage, stomach lavage, cloacal fluid, and blood clots of the turtles studied, demonstrating that captive *Kinosternon scorpioides* turtles in the Amazon region were exposed to a pathogenic *Leptospira* of the P1 subclade.

## Figures and Tables

**Table 1:** Distribution of biological samples collected and results of the molecular analysis for *Leptospira* spp. DNA in *Kinosternon scorpioides* in the Amazon Biome.

Sample	Collected	Positives	Negatives	Sequenced
Cloacal Lavage	40	4	36	2
Stomach Lavage	40	1	39	0
Cloacal fluid	27	3	24	0
Blood clot	40	11	29	9
TOTAL	147	19	128	11

**Table 2:** PCR results for DNA detection of *Leptospira* spp. in cloacal lavage, stomach lavage, cloacal fluid, and blood clots from *Kinosternon scorpioides*.

ID	Analysis Result			
	Blood Clot	CL	SL	Cloacal Fluid
01	-	-	+	Nc
02	-	-	-	Nc
03	-	-	-	Nc
04	-	-	-	+
05	-	-	-	Nc
06	-	-	-	Nc
07	-	-	-	Nc
08	-	-	-	-
09	-	-	-	Nc
10	-	+	-	-
11	+	+	-	-
12	-	-	-	-
13	+	-	-	+
14	-	-	-	+
15	-	-	-	-
16	-	-	-	-
17	-	-	-	Nc
18	+	-	-	Nc
19	-	-	-	-
20	-	-	-	-
21	+	-	-	-
22	-	-	-	Nc
23	-	-	-	-
24	+	-	-	-
25	+	-	-	-
26	-	-	-	-
27	-	-	-	-
28	-	-	-	-
29	-	-	-	-
30	-	-	-	-
31	+	-	-	-
32	-	-	-	-
33	+	-	-	Nc
34	-	-	-	Nc
35	+	-	-	-
36	+	-	-	-
37	-	-	-	-
38	-	-	-	-
39	+	+	-	-
40	-	+	-	Nc

Identification; Nc: not collected; CL: cloacal lavage; SL: stomach lavage; positive samples, +; negative samples, -.

## Data Availability

The original contributions presented in the study are included in the article.
